# Statistical Methods for Item Reduction in a Representative Lifestyle Questionnaire: Pilot Questionnaire Study

**DOI:** 10.2196/28692

**Published:** 2022-03-18

**Authors:** Alessio Staffini, Kento Fujita, Akiko Kishi Svensson, Ung-Il Chung, Thomas Svensson

**Affiliations:** 1 Precision Health, Department of Bioengineering Graduate School of Engineering The University of Tokyo Tokyo Japan; 2 Department of Economics and Finance Catholic University of Milan Milan Italy; 3 Data Solutions Division Project Promotion Department Albert Inc Tokyo Japan; 4 Data Service Infrastructure Development Department, Service Infrastructure Division, IT-OT Innovation Division Mobile Technology Unit SoftBank Corp Tokyo Japan; 5 Department of Clinical Sciences Lund University Malmö Sweden; 6 Department of Diabetes and Metabolic Diseases The University of Tokyo Tokyo Japan; 7 School of Health Innovation Kanagawa University of Human Services Kawasaki Japan; 8 Clinical Biotechnology Center for Disease Biology and Integrative Medicine, Graduate School of Medicine The University of Tokyo Tokyo Japan

**Keywords:** item reduction, surveys and lifestyle questionnaires, feedback measures, questionnaire design, variance inflation factor, factor analysis, mobile phone

## Abstract

**Background:**

Reducing the number of items in a questionnaire while maintaining relevant information is important as it is associated with advantages such as higher respondent engagement and reduced response error. However, in health care, after the original design, an *a posteriori* check of the included items in a questionnaire is often overlooked or considered to be of minor importance. When conducted, this is often based on a single selected method. We argue that before finalizing any lifestyle questionnaire, *a posteriori* validation should always be conducted using multiple approaches to ensure the robustness of the results.

**Objective:**

The objectives of this study are to compare the results of two statistical methods for item reduction (variance inflation factor [VIF] and factor analysis [FA]) in a lifestyle questionnaire constructed by combining items from different sources and analyze the different results obtained from the 2 methods and the conclusions that can be made about the original items.

**Methods:**

Data were collected from 79 participants (heterogeneous in age and sex) with a high risk of metabolic syndrome working in a financial company based in Tokyo. The lifestyle questionnaire was constructed by combining items (asked with daily, weekly, and monthly frequency) from multiple validated questionnaires and other selected questions. Item reduction was conducted using VIF and exploratory FA. Adequacy tests were used to check the data distribution and sampling adequacy.

**Results:**

Among the daily and weekly questions, both VIF and FA identified redundancies in sleep-related items. Among the monthly questions, both approaches identified redundancies in stress-related items. However, the number of items suggested for reduction often differed: VIF suggested larger reductions than FA for daily questions but fewer reductions for weekly questions. Adequacy tests always confirmed that the structural detection was adequate for the considered items.

**Conclusions:**

As expected, our analyses showed that VIF and FA produced both similar and different findings, suggesting that questionnaire designers should consider using multiple methods for item reduction. Our findings using both methods indicate that many questions, especially those related to sleep, are redundant, indicating that the considered lifestyle questionnaire can be shortened.

## Introduction

### Background

Short questionnaires have several advantages over long ones: they are less expensive to design [1]; are associated with less random or systematic error or noise in the reported results caused by lack of motivation, fatigue, or boredom [2-4]; and have higher respondent engagement, with empirical evidence indicating that long questionnaires are associated with a low response rate [5]. Therefore, reducing the number of survey items is important.

A number of approaches to reduce survey items have been studied. First, in psychometrics, the Cronbach α coefficient is often used in Classical Test Theory [6] to assess the internal consistency of a given questionnaire. Second, factor analysis (FA) and principal component analysis were used to extract a latent structure and explain most of the data variance with fewer factors or components than the original questionnaire items (eg, the studies by McHorney et al [7], Bai et al [8], and Brosnan et al [9]). Third, researchers have also focused on Item Response Theory (IRT) [10,11]; however, IRT methods are most effective when the original questionnaire or survey is developed using IRT or when there are theoretical reasons to expect it to fit an IRT model [12]. Recent studies have used the variance inflation factor (VIF) to better address collinearity problems among covariates when performing regression analysis of survey data [13]. However, despite the advantages and practicality of automatic variable selection, simulations suggest that the VIF does not necessarily identify a true underlying model [14]. In general, it is better not to rely on a single method for variable reduction and instead to compare the results of multiple approaches.

Although it is important to minimize errors in questionnaire responses in all fields, it is paramount in medicine and public health, where omissions and inaccuracies can lead to possible misdiagnoses and subsequent incorrect treatments or interventions. Despite this, emphasis in health care is often placed only on the initial design, where a questionnaire is typically evaluated by comparing its internal consistency with that of other similar questionnaires or with a different version of the same questionnaire [15-17]. The questionnaires also sometimes include open-ended questions, despite evidence showing that such questions often do not provide sufficiently solid insights [18]. Although an *a priori* carefully designed questionnaire is extremely important, we believe that including an *a posteriori* check of the questions before making any result-based inferences will help improve the overall quality of the instrument and should, therefore, become part of standard procedure.

### Objective

Our study builds upon other research conducted in this direction: to name a few related works, Cappelleri et al [19] applied FA to develop a questionnaire to measure the satisfaction of patients with type 1 diabetes, highlighting that the two key factors were convenience and social comfort; Juniper et al [20] compared the results of the impact method, which preserves items according to their relative importance as perceived by the patients, with the results of FA in a quality of life questionnaire, suggesting that different approaches lead to different results; Arifin and Yusoff [21] applied confirmatory FA, as well as performed other statistical tests, for an emotional intelligence inventory to be used among medical course applicants. In the Classical Test Theory branch, researchers often compared Cronbach α with Rasch analysis; for example, Prieto et al [22] did so on a 38-item health questionnaire, concluding that the methods led to similar results; Erhart et al [23] analyzed the possibility of performing item reduction by comparing the results of Cronbach α with those of Rasch item fit for a health-related quality of life questionnaire administered to children and adolescents, concluding, on the other hand, that both methods should be accompanied by additional analyses.

We found that very often, researchers focused on a single technique or similar approaches without comparing the results from multiple different methods. This was the starting point of our study. Despite its desirable properties (such as automatic identification and remotion of multicollinear items) and statistical foundations, VIF is rarely directly used as an item reduction technique. FA indirectly deals with the same multicollinearity problem by grouping *similar* variables under the same factor; however, its inner logic is different, as multicollinearity is solved because of dimensionality reduction. To the best of our knowledge, this is the first study to directly compare how similar or how different the results obtained from these methods are when applied to the items of a lifestyle questionnaire.

The aim of this study is to compare the results of VIF and FA for item reduction in a lifestyle questionnaire constructed by combining items from different sources, with questions asked with daily, weekly, or monthly frequency.

## Methods

### Study Participants

The collected questionnaire data (described in detail in the following sections) form part of a 1-month pilot study for lifestyle interventions, with a more general aim than the one described in this paper; that is, in particular, to investigate the feasibility of using a smartphone lifestyle intervention app and assess the feasibility of collecting data from a wearable device. The recruited participants were individuals with a high risk of metabolic syndrome. The sample size was calculated based on accepting an α risk of .05 and a β risk of .25 in a 1-tailed test, with an estimated 10% loss to follow-up. The estimated sample size was 120 participants. A total of 117 individuals consented to participate; however, 2 (1.7%) individuals were withdrawn, leaving a total of 115 (98.3%) participants. The 115 participants were randomly allocated into 2 groups: the intervention group (79/115, 68.7% individuals who followed a lifestyle education program over the study period) and the control group (36/115, 31.3% individuals). Allocation to the intervention and control groups occurred through randomization, with stratification by sex and age (<40 years or >40 years). Participants in the intervention group (the focus of this study), men and women aged between 29 and 58 years, were selected from a financial company based in Tokyo. To minimize sampling bias in the collected questionnaire data, we confirmed that the intervention group was sufficiently heterogeneous in terms of age, sex, and employment conditions. The initial study excluded those who had a history of serious medical conditions, had received any other lifestyle intervention, planned to take long vacations, had night shifts, and were pregnant (or those with suspected pregnancy). The study period of the research presented in this paper was from March 2 to March 30, 2018.

All research was performed in accordance with relevant ethical guidelines and regulations. All the participants received detailed information about the purpose of the study in writing and during explanatory face-to-face meetings. All participants provided written informed consent and understood that participation was completely voluntary and could be discontinued at any time without any disadvantage or penalty. Participants were given a wearable device as an incentive for participation.

### Questionnaire

The intervention group participants were asked to respond to lifestyle-related questions through a smartphone-based mobile app for a month. For the purposes of this study, we included participants who answered any of the questions.

We constructed a lifestyle questionnaire comprising 51 questions, focusing in particular on the domains of sleep, stress, nutrition, and alcohol and tobacco consumption. For brevity, the questionnaire items are indicated by *item X,* where *X* represents the item number. The questionnaire is provided in Multimedia Appendix 1.

Daily questions on sleep quality were selected from the validated St Mary’s Hospital Sleep Questionnaire (items 2-5) [24]. Daily questions to assess dietary habits were taken from the Dietary Guidelines provided by the Ministry of Agriculture, Forestry, and Fisheries of Japan (Food Safety and Consumer Affairs Bureau; items 6-16). Daily questions about caffeine intake (items 17 and 18) and stress levels (items 20 and 21) have been widely used in many population-based cohort studies. In addition, we added 2 other daily questions, not taken from any validated questionnaire, about opening the smartphone-based app in the morning (item 1) and alcohol consumption (item 19). Most of the weekly questions (21/26, 81%) were taken from the validated General Sleep Disturbance Scale (items 25-45) [25]. We added 5 other questions to assess the participants’ commitment to reducing alcohol intake (item 47), determine the number of cigarettes smoked per day over the past week and interest in a smoking cessation program (items 23 and 46), and information about working conditions (items 22 and 24). Finally, monthly questions were taken from the validated Perceived Stress Scale–4 (items 48-51) [26]. As shown in Multimedia Appendix 1, items 48 and 51 are reverse-coded items; this is sometimes recommended to cross-check the validity of the responses.

When a participant did not complete a daily questionnaire item, the data were treated as missing and excluded from the analysis. However, when a participant did not complete a weekly or monthly questionnaire item, we assumed that their subsequent response to the item was applicable for the entire respective week or month and, thus, backfilled missing values.

### Statistical Analyses

#### Overview

All statistical analyses were performed using Python (version 3.7.4) on the Anaconda platform (Anaconda Inc). To perform effective item reduction, it is important to verify that the variables we want to exclude, in fact, explain the same underlying variability as the other variables remaining in the questionnaire. We considered two statistical approaches: VIF and FA.

#### VIF Analysis

VIF is the quotient of the variance in a model with multiple variables and the variance in a model with only 1 variable. It indicates the strength of multicollinearity among a set of variables, assuming that they have a linear relationship. Each variable, in turn, is regressed on all other variables present in the set. Considering a set *A* of *n* variables, the VIF associated with variable *i ∈ A* is defined as follows:




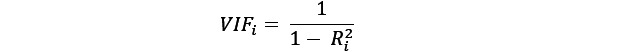




Here, *R_i_^2^* is the coefficient of determination obtained by ordinary least squares regression and regressing variable *i* on all the other *j ≠ i* variables in the set. A higher *VIF_i_* reportss greater collinearity between variable *i* and the other predictors. As 0≤*R_i_^2^*≤1, *VIF_i_∈ [1, ∞)*.

There is no general consensus on the ideal VIF threshold for indicating multicollinearity, with many different values having been used in the empirical literature. For example, Vittinghoff et al [27] suggested a threshold value of 10, whereas Johnston et al [28] were more conservative and used a threshold value of 2.5. Keeping in mind that multicollinearity is a bigger problem with a small sample size [29], we selected a threshold value of 5; this cutoff is often selected to establish a high *risk* of multicollinearity [30,31].

We applied VIF iteratively: first, we calculated *VIF_i_* for each variable in set *A*; then, we removed the variable with the highest calculated VIF value (as it is already well-explained by the remaining variables) and recalculated VIF for each remaining variable. The process was stopped when all remaining variables had a calculated VIF ≤5 so that there was no concern of high collinearity among the variables.

#### FA Method

FA is a statistical method used to describe variability among observed and correlated variables in terms of a fewer number of unobserved and underlying variables called factors. This method aims to identify the independent factors that explain the different sources of variability in the original variables. It assumes that any observed variable is directly associated with an underlying factor. Informally, FA shares many similarities with principal component analysis: they are both data analysis techniques, their goal being to reduce a large number of variables into a fewer number of more treatable and interpretable new variables, trying to minimize the information loss at the same time. This dimensionality reduction is performed by projecting the original data onto a lower-dimensional space, preserving as much variability as possible. More formally, consider a set *A* of *n* observable variables for which we assume that each variable *x_i_* can be expressed as a linear combination of *k*<*n* factors and intercept *β_i_*:

x_i_ = β_i_ + l_i1_F_1_ + l_i2_F_2_ + ... + l_ik_F_k_ + ε_i_

where *ε_i_* ~ N (0, σ^2^). We can rewrite the above in matrix notation:

*X* = *β* + *LF* + ε,

where *X*=(*x_1_*,…,*x_n_*)*^T^*, *β*=(*β_1_*,…,*β_n_*)*^T^*, and 
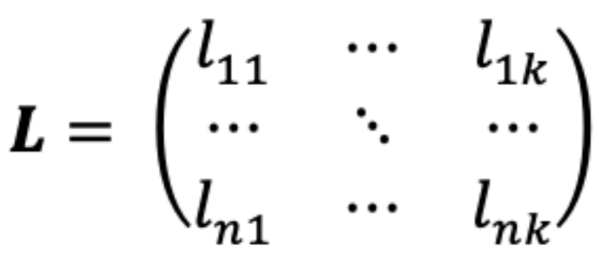
 denote the factor-loading matrix, *F*=(*F_1_*,…,*F_k_*)*^T^* is the vector of common factors, and *ε*=(*ε_1_*,…,*ε_n_*)*^T^* is the vector of unobserved error terms. The factor-loading matrix *L* expresses the relationship of each observed variable with the unobserved factors, showing the variance explained by each observed variable for each factor. If we consider *X* *∈* *R^n^* to be the vector representing all questionnaire items, we can replace it with the vector *F* *∈* *R^k^* of unobserved factors, which lies in a lower-dimensional space. We can think of each factor as being capable of explaining a certain variance in the original items, and we can further exclude factors with the lowest amount of explained variance (the criterion for selecting the number of factors is explained in the following sections).

We looked for joint variations in the observed variables in response to unobserved latent factors. Three assumptions should be satisfied: there should be no outliers in the data, the sample size should be *big enough*, and there should be no perfect multicollinearity between the observed variables [32]. Our data set satisfied all of these assumptions.

We further applied two adequacy tests before proceeding with FA: the Bartlett test of sphericity [33] and the Kaiser–Meyer–Olkin (KMO) test [34]. The Bartlett test of sphericity checks whether the observed variables are effectively correlated with each other by comparing their correlation matrix *corr(X)* against the identity matrix *I*. If the null hypothesis (*corr(X)*=*I*) cannot be rejected, this indicates that the original variables are orthogonal (hence, unsuitable for structure detection), and any data reduction technique (such as FA) would not produce any meaningful result. The KMO test is another way of measuring the suitability of data for FA; it estimates the proportion of variance that may be common (ie, caused by the same underlying factor) among all observed variables, with a lower proportion being a more suitable condition for FA. KMO values range between 0 and 1 (according to the original paper by Kaiser [34], values of a statistic <0.5 mean that performing FA is not adequate). Together, the 2 adequacy tests check the data distribution and sampling adequacy.

Finally, we used the Kaiser criterion [35] to select an adequate number of factors. The eigenvalues *λ_i_* of the correlation matrix *corr(X)* can be used to measure the degree to which the factors explain the variance in the observed variables. Thus, any factor with an associated eigenvalue *λ_i_*>1 explains more of the variance than a single (observed) variable. As a selection criterion, the number of factors was chosen such that it was equal to the number of eigenvalues >1. However, the Kaiser criterion has been criticized for being an arbitrary approach [36]. Thus, we further examined the scree plot of the eigenvalues and checked whether our stopping criterion matched the possible points of inflection. Finally, we used ordinary least squares to identify the minimum residual solution, which is also the default method for exploratory FA, when estimating the factor loadings.

### Ethical Approval

This study was approved by the research ethics committee of the Faculty of Medicine, University of Tokyo (application number 11781).

## Results

For meaningful comparisons, we grouped the questions according to the frequency at which they were asked (daily, weekly, and monthly) and analyzed each group separately.

### Daily Questions

A total of 21 questions were asked daily to 79 users for a total of 1746 collected data points. After excluding missing values, of the 1746 collected data points, 1658 (95%) data points were left for analysis.

As described in the *Methods* section, we iteratively excluded all the variables for which *VIF_i_*>5. By doing so, the 21 initial variables were reduced to 3 (items 4, 20, and 21).

**Figure 1:**
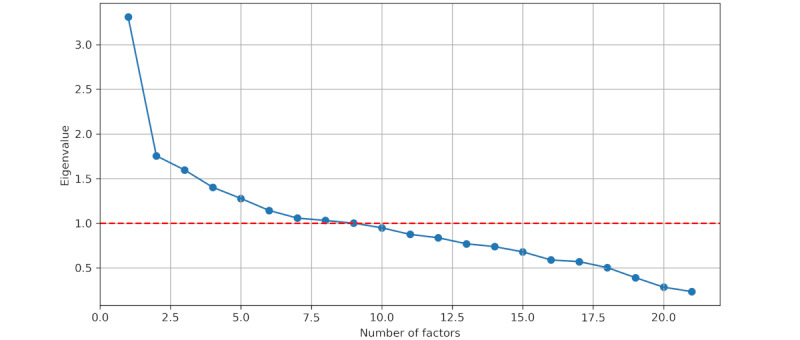
Eigenvalues scree plot for daily questions.

When FA was applied to the daily questions set, we obtained a *P* value of 0 using the Bartlett test; thus, we rejected the null hypothesis *corr(X)*=*I* at all significance levels, confirming that the considered variables were correlated with each other. The KMO test yielded a value of 0.73, further confirming that the structure detection was adequate for the considered variables. The Kaiser criterion led to the selection of 9 factors (with the first factor having a significantly higher eigenvalue), as shown in the eigenvalue scree plot in Figure 1.

By analyzing the factor-loading matrix *L* of the 9 factors for daily questions, we observed the following: (1) factor 1 had high factor loadings for items 2, 3, 4, and 5 (sleep-related questions); (2) factor 2 had high factor loadings for item 20 (stress level); (3) factor 3 had high factor loadings for item 6 (whether eating 3 times a day); (4) factor 4 had high factor loadings for items 16, 17, and 18 (related to caffeinated drinks); and (5) factor 5 had high factor loadings for items 9 and 12 (having lunch in a restaurant or having a takeout lunch). The other factors were less interesting (eg, as they were each associated with 1 or 2 variables only and displayed factor loadings smaller in magnitude than those of the 5 abovementioned factors) or were more difficult to interpret, as the amount of variance they explained was relatively low and spread across multiple variables. The factor-loading matrix for daily questions is provided in Multimedia Appendix 2.

When the sample size is small (typically <300, as in our analysis), it is also worth looking at the average communality of the retained items [37]. Using 9 underlying factors, we obtained an average communality of 0.492, which is an acceptable value when using Promax rotation [38], as we did for our analysis.

For the main 5 abovementioned factors, we also conducted a reliability analysis using Cronbach α to measure the internal consistency of our underrepresentation. The α coefficient for factor 1 was high (.895), indicating high internal consistency for the sleep-related items, confirming the reliability of the factor. Factors 2 and 3 had high factor loadings for a single variable; thus, we could not check any interitem internal consistency. Factors 4 and 5 displayed lower α coefficients (.470 and .536, respectively), which might be a consequence of the low number of represented original items (3 and 2, respectively) than factor 1 (4), as well as a reduced scale with respect to the sleep-related questions.

The 9 underlying factors (instead of the 21 observed variables) explained 49.25% of the total variance.

### Weekly Questions

A total of 26 questions were asked weekly to 79 users. The VIF approach reduced the initial 26 questions to 21 (excluding items 28, 30, 35, 46, and 47).

**Figure 2:**
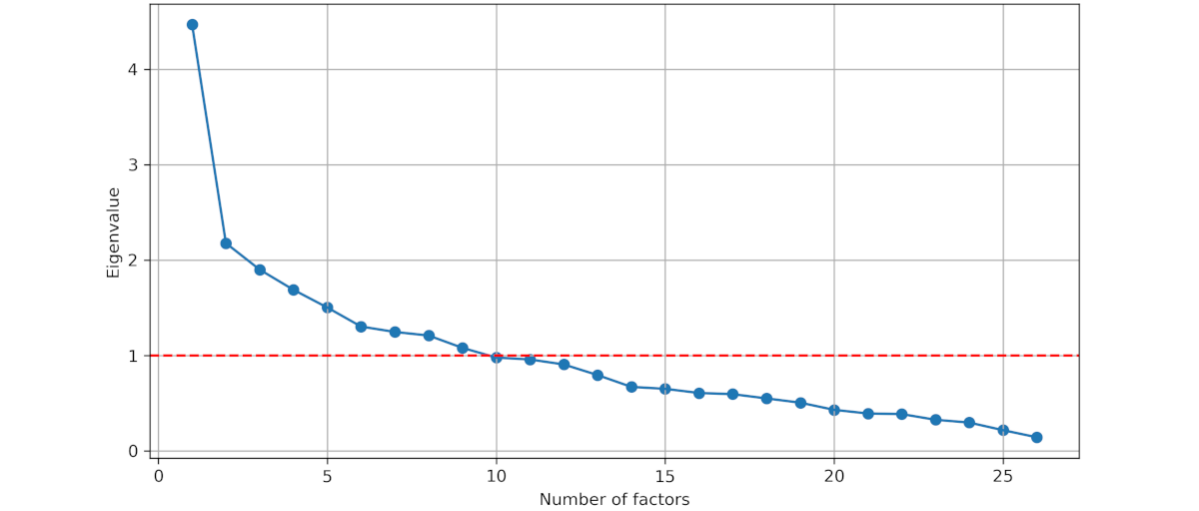
Eigenvalues scree plot for weekly questions.

When applying FA to the weekly question set, we again obtained a *P* value of 0 using the Bartlett test, and the KMO test produced a value of 0.69. The Kaiser criterion led to the selection of 9 factors. Similar to the eigenvalues for the daily questions, the first factor exhibited a significantly higher eigenvalue (Figure 2).

By analyzing the factor-loading matrix *L* of the 9 factors for weekly questions, we observed the following: (1) factor 1 had high factor loadings for items 26, 30, 31, and 39 (sleep-related questions); (2) factor 2 had high factor loadings for items 28, 34, and 35 (questions related to satisfaction and feeling well); (3) factor 3 had high factor loadings for items 32 and 33 (feeling annoyed and feeling tired during the day); (4) factor 4 had high factor loadings for items 22 and 24 (weekly number of days off and weekly hours of work); and (5) factor 5 had high factor loadings for items 23 and 46 (amount of tobacco consumption and degree of interest in a smoking cessation program). The other factors were less interesting (eg, as they were each associated with 1 or 2 variables only and displayed factor loadings smaller in magnitude than those of the 5 abovementioned factors) or were more difficult to interpret, as the amount of variance they explained was relatively low and spread across multiple variables. The factor-loading matrix for weekly questions is provided in Multimedia Appendix 3.

Using 9 underlying factors, we obtained an average communality of 0.493, which is acceptable.

For the main 5 abovementioned factors, the results of the reliability analysis indicated high or acceptable Cronbach α coefficients for all considered factors (.757, .821, .721, .679, and .609), further confirming the internal consistency of the considered underrepresentation.

The 9 underlying factors (instead of the 26 observed variables) explained 49.29% of the total variance.

### Monthly Questions

A total of 4 questions were asked monthly to the 79 users. The VIF approach reduced the initial 4 questions to 2 (leaving items 50 and 51).

**Figure 3:**
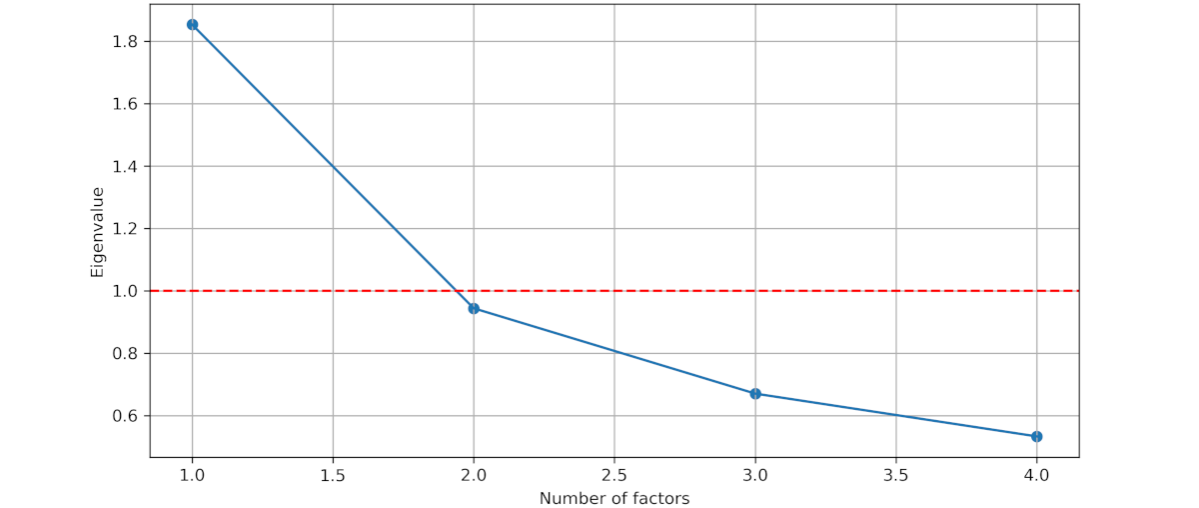
Eigenvalues scree plot for monthly questions.

When applying FA to the monthly question set, we again obtained a *P* value of 0 using the Bartlett test, and the KMO test produced a value of 0.64. The Kaiser criterion led to the selection of a single factor (Figure 3).

By analyzing the factor-loading vector *L* of the factor for monthly questions, we observed that factor 1 had relatively high factor loadings for all 4 monthly questions, in particular items 50 and 51 (*degree of feeling on top of things* and *degree of not being able to cope with all the things that needed to be done*, respectively). The factor-loading vector for monthly questions is provided in Multimedia Appendix 4.

Using a single underlying factor, we obtained an average communality of 0.308, which is too low. However, as communalities are the proportion of each variable’s variance that can be explained by the factors (in this case, a single factor), it makes sense that their average matches the cumulative variance explained by the single factor itself.

For the single considered factor, the results of the reliability analysis indicated an acceptable Cronbach α coefficient (.607), further confirming the internal consistency of the considered underrepresentation.

The single underlying factor (instead of the 4 observed variables) explained 30.76% of the total variance.

## Discussion

### Principal Findings

The objective of this study was to compare 2 different statistical methods, which are often used as reduction techniques, and their results in a lifestyle questionnaire, which was constructed using a variety of questions (asked with daily, weekly, and monthly frequency) aimed at evaluating the general well-being of an individual. Our main findings suggest not only that existing validated lifestyle questionnaires might benefit from further item reduction (in the questions about sleep quality and satisfaction, in particular) but also that different algorithms lead to different results for what concerns other groups of items.

Indeed, the results obtained using the two considered methods (VIF and FA) shared some similarities but also exhibited substantial differences.

Among daily questions, VIF led to the exclusion of many more variables than FA, retaining only 1 question about sleep and 2 questions aimed at evaluating stress levels at work and at home, respectively. Although we expected the sleep items to be highly correlated, the iterative approach of deleting correlated variables also led to the exclusion of all items on eating habits, suggesting that, at least in our data sample, these variables shared some correlation with sleep satisfaction and stress level components. The two types of stress do not seem to be well correlated, suggesting that stress at home does not depend on the amount of stress felt at work and vice versa. FA suggested the inclusion of only 9 factors instead of the original 21 variables; however, in line with the VIF, the factor with the highest eigenvalue captured the variance of the sleep-related items.

Among the weekly questions, the VIF reduced the initial 26 weekly questions to 21 questions. Among the 5 deleted questions, 3 (60%) were sleep-related, and it made sense that they were correlated with the other weekly sleep items. However, interestingly, there was also a correlation between desiring a healthier lifestyle (identified as an interest in quitting smoking and consideration for reducing alcohol intake) and work habits or sleep satisfaction level. This is in line with findings from Hidaka et al [39], who observed a positive correlation between low sleep satisfaction and unhealthy lifestyle patterns in the Japanese population. FA produced similar results, identifying 1 factor in particular for sleep-related questions and another factor for general well-being.

Finally, the monthly questions selected from the Perceived Stress Scale–4 aimed to assess different aspects of the consequences of stress. For example, stress is known to negatively affect self-confidence [40] and, in the long-run, also mental and physical health [41]. Therefore, we expected that the analyses would lead to the exclusion of a significant number of these items. Indeed, the VIF reduced the initial 4 questions to 2, and the FA identified a single significant factor.

We observed that despite the fact that both methods deal with the same underlying problem of multicollinearity, VIF led to greater item reduction in some instances, whereas FA did so in others. Thus, we suggest that questionnaire designers use both methods and, in the event of a discrepancy in results, adopt other additional measures such as comparing both results with the consistency of the internal questionnaire obtained using Cronbach α for the final selection.

As previously mentioned, our questionnaire included 2 reverse-coded questions. In our methods, we analyzed the absolute value of the correlation coefficients to eliminate the need for any additional operations to identify the direction of the correlation (positive or negative).

The principal result of our study shows that even in validated lifestyle questionnaires, many items (particularly sleep-related ones) are indeed redundant. Therefore, when aiming for short questionnaires, we suggest that questionnaire designers should always consider the application of item reduction instruments after a trial phase, as certain items could, in principle, be deleted without incurring significant information loss.

Our study had some limitations that warrant mention. First, because of factor loadings, FA can also be used to analyze the amount of information that is lost when switching from the original variables to the underlying factors. In general, it should be noted that the exclusive use of statistical methods to shorten questionnaires can lead to the loss of valuable information [42]. Indeed, this was observed in our results; the total variance explained exclusively by the identified factors never exceeded 50%. Therefore, rather than providing a recipe that is indiscriminately valid, our approach was to focus on identifying areas in which there is a high possibility of reducing questionnaire items with as little information loss as possible. Our empirical results show that sleep-related questions are, by far, the area where such a reduction seems the most feasible. Second, as this was a pilot study, the number of participants was relatively small. This is the major limitation of our study, as a bigger data set would provide more statistically sound results. However, despite its small size, the size of our data set was considered acceptable for the analyses we conducted [43,44]. Furthermore, despite differences in age and sex, the participants were all selected from the same company, which could have introduced some selection bias. Thus, the results obtained in this study should be validated in further studies.

### Conclusions

We constructed a lifestyle questionnaire by combining items from various authoritative sources. We then applied two different statistical methods for item reduction (VIF and FA) to check whether the existing items in the three groups of questions (asked with daily, weekly, and monthly frequency) were redundant. The results of the applied methods did not always match but nevertheless provided evidence that many items related to sleep, in particular, were indeed redundant. Two reduced questionnaires (according to VIF and FA) are proposed in Multimedia Appendix 5. We also conducted reliability analyses for each group of questions using Cronbach α to measure the consistency of the obtained underrepresentation, obtaining satisfactory results. Our results suggest that questionnaire designers should always conduct a *trial* phase on a sample of participants, and examine the correlation between the items, before finalizing any lifestyle questionnaire.

## Appendix

Multimedia Appendix 1 Lifestyle questionnaire.

Multimedia Appendix 2 Factor loading matrix for daily questions.

Multimedia Appendix 3 Factor loading matrix for weekly questions.

Multimedia Appendix 4 Factor loading vector for monthly questions.

Multimedia Appendix 5 Proposed reduced lifestyle questionnaires.

## Abbreviations

FA: factor analysis
IRT: Item Response Theory
KMO: Kaiser–Meyer–Olkin
VIF: variance inflation factor
