# Supplementary figures and images for: Statistical Methods for Item Reduction in a Representative Lifestyle Questionnaire: Pilot Questionnaire Study
Source: Interact J Med Res. 2022 Mar 18;11(1):e28692. doi: 10.2196/28692 (PMC8976253; doi:10.2196/28692)

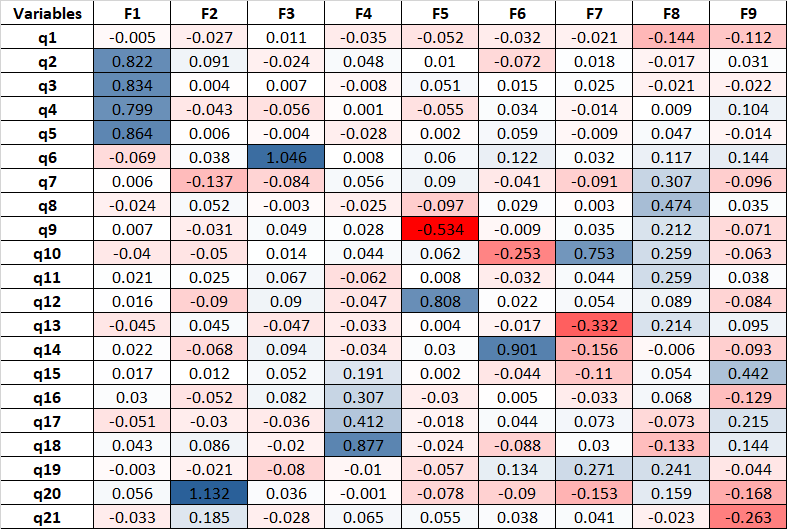

Supplement: Multimedia Appendix 2 [file ijmr_v11i1e28692_app2.png]

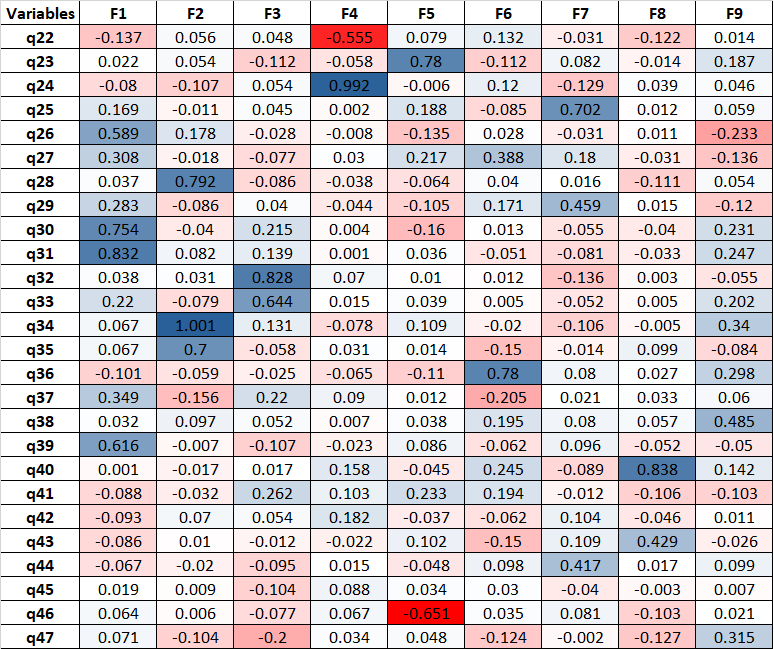

Supplement: Multimedia Appendix 3 [file ijmr_v11i1e28692_app3.png]

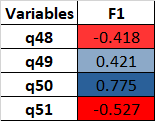

Supplement: Multimedia Appendix 4 [file ijmr_v11i1e28692_app4.png]
